# Rapid-Onset Obesity Due to Impulsive Food-Seeking Behavior in a Puerto Rican Child With CHRNA7 15q13.3 Microdeletion

**DOI:** 10.7759/cureus.14012

**Published:** 2021-03-20

**Authors:** Brian L Jiménez, Simón Carlo, Wilfredo De Jesús Rojas

**Affiliations:** 1 Pediatrics, Ponce Health Sciences University - School of Medicine, Ponce, PRI; 2 Genetics, Ponce Health Sciences University - School of Medicine, Ponce, PRI; 3 Pediatric Pulmonology, University of Puerto Rico - Medical Sciences Campus, San Juan, PRI; 4 Pediatric Pulmonology, Ponce Health Sciences University - School of Medicine, Ponce, PRI; 5 Pediatric Pulmonology, San Juan Bautista School of Medicine, Caguas, PRI

**Keywords:** chrna7, 15q13.3 deletion, hyperphagia, rapid-onset obesity, developmental delay, behavioral disorder

## Abstract

A microdeletion in the 15q13.3 locus is an exceedingly rare condition affecting the CHRNA7 gene. There have been 11 pediatric cases of this mutation reported worldwide. Clinical characteristics of the 15q13.3 microdeletion are rapid-onset obesity, hypotonia, autism, seizures, congenital cardiac defects, and neuropsychiatric disorders including impulsive hyperphagia. We describe the case of a four-year-old female with CHRNA7 15q13.3 microdeletion presenting with morbid obesity due to impulsive food-seeking behavior. We have also conducted a literature review on 15q13.3 microdeletion and compared the clinical features with other rapid-onset obesity disorders in the pediatric population. The goal of this case report is to increase awareness concerning CHRNA7 15q13.3 microdeletion as part of the differential diagnosis of rapid-onset obesity associated with neuropsychiatric disorders in pediatrics.

## Introduction

The CHRNA7 gene is responsible for encoding the α7 nicotinic acetylcholine receptor and is expressed in both the peripheral and central nervous systems [1]. CHRNA7 copy number variants have been linked to multiple cognitive and behavioral disorders, including attention deficit hyperactivity disorder (ADHD), autism spectrum disorder (ASD), epilepsy, schizophrenia, and bipolar disorder [1]. Deletions along the CHRNA7 gene, particularly 15q13.3 deletions, have been associated with severe clinical manifestations [2].

The predominant findings associated with 15q13.3 microdeletions were related to neuropsychiatric conditions, primarily developmental delay and intellectual disability. Less common findings are congenital malformations, with the most common being congenital heart disease [3]. We describe the case of a pediatric patient with CHRNA7 15q13.3 microdeletion with morbid obesity of rapid-onset due to impulsive food-seeking behavior. This article was previously presented as a poster at the Puerto Rico Pediatric Society: 68th Annual Congress on February 20, 2021.

## Case presentation

**Figure 1:**
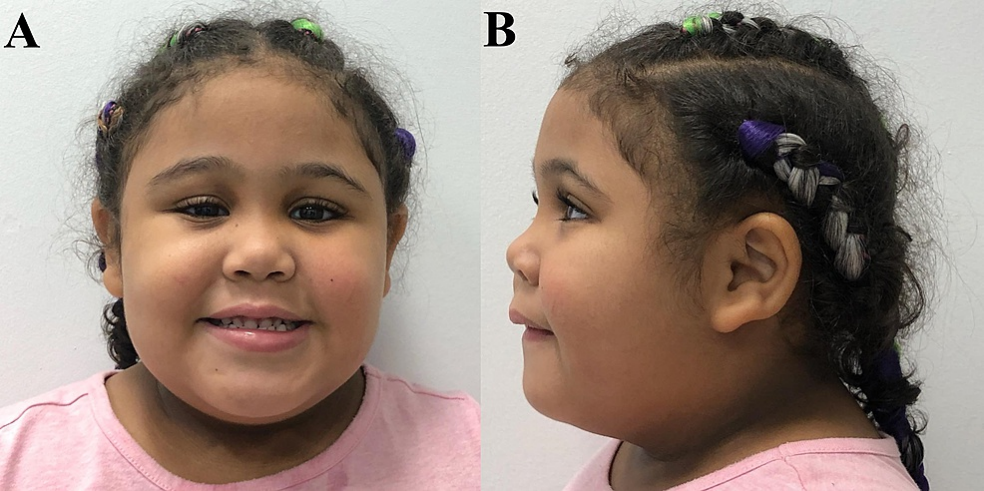
Facial features in our Puerto Rican CHRNA7 15q13.3 microdeletion case. (A): Anterior view of patient’s face notable for congenital ptosis of the right eyelid. (B): Lateral view displays broad nasal bridge and velvety discoloration around her neck.

**Figure 2:**
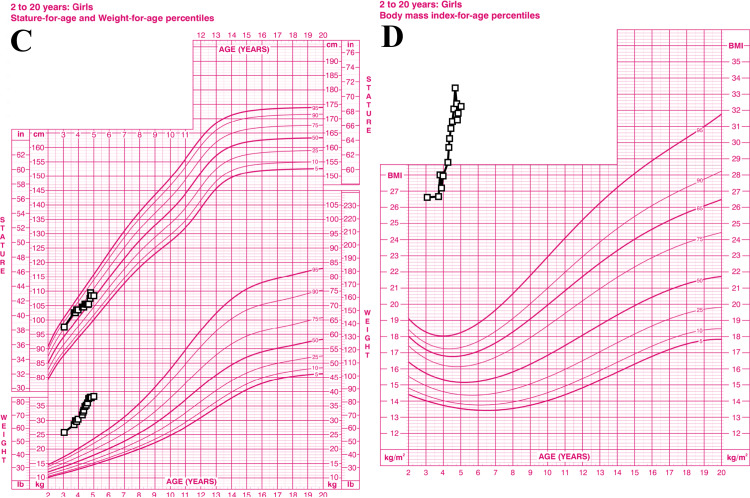
Graphical representation of the patient’s growth. (C): Superior data points represent the patient’s height, while inferior points represent her weight. (D): Graph of same patient’s BMI, which is well above the 99.9th percentile for age and sex.

The case of a four-year-old Puerto Rican female with a history of speech and motor delay, impulsive food-seeking behavior, and rapid-onset obesity since the age of two. As per the caretaker report, abnormal eating behaviors included: stealing food from others in a restaurant, eating uncooked food from the pantry and fridge, and ingesting food remnants from the garbage can. On physical examination, the patient was noted to have congenital ptosis of the right eyelid, broad nasal bridge, and velvety discoloration around her neck (Figure 1). Marked morbid obesity was also present on physical examination with a BMI above the 99.9th percentile for age and sex (Figure 2). Genetic evaluation and sleep studies ruled out other disorders associated with rapid-onset obesity including Prader-Willi syndrome and Rapid-onset Obesity with Hypothalamic Dysregulation, Hypoventilation, and Autonomic Dysregulation (ROHHAD) syndrome. Chromosomal microarray revealed a microdeletion of 443 kb at the 15q13.3 locus which explains the etiology of her phenotype and impulsive behavior. A multidisciplinary care approach was initiated for weight control and management of her additional comorbidities. Familial genetic studies were not completed in this case.

## Discussion

The 15q13.3 microdeletion syndrome is an exceedingly rare condition with a varied clinical presentation and few reported cases, with only 11 patients currently described in the literature. The various modes of regulation, multiple functions, and wide expression of CHRNA7 create significant difficulty when studying this gene [1]. The multiple neuropsychiatric conditions presenting as a result of this mutation can be difficult to distinguish from other clinical syndromes [2]. For this reason, it is especially important to differentiate the signs and symptoms from some of the syndromes that most closely resemble 15q13.3 microdeletion, namely Prader-Willi and ROHHAD syndrome (Table 1). Some of the associated diagnoses include ADHD, ASD, intellectual disability, epilepsy, schizophrenia, and bipolar disorder [3]. Neurological evaluation is also likely to reveal vision impairment, hypotonia, and abnormal electroencephalogram (EEG) findings [3].

**Table 1:** Comparison of clinical features in our case with others previously reported in the literature. ROHHAD: Rapid-onset Obesity with Hypothalamic Dysfunction, Hypoventilation, and Autonomic Dysregulation; PW-like: Prader-Willi-like; ADHD: Attention-deficit hyperactivity disorder; EEG: Electroencephalogram.

Clinical Feature	15q13.3 Microdeletion	Prader-Willi Syndrome	ROHHAD
PW-like features (obesity)	X	X	X
Hyperphagia	X	X	X
Mild dysmorphic features	X	X	
Intellectual disability	X	X	X
Developmental delay	X	X	X
Speech/Language impairment	X	X	X
Autism spectrum disorder	X	X	X
ADHD	X	X	X
Schizophrenia	X	X	
Multiple congenital abnormalities	X		
Congenital heart disease	X		
Hypotonia	X	X	
Seizures	X	X	X
EEG abnormalities	X		
Visual impairment	X		
Genital hypoplasia		X	
Growth hormone deficiency		X	X
Hypothyroidism			X
Hyperprolactinemia			X
Hypoventilation			X

At present, there are no specific guidelines or algorithms in place to standardize the treatment of this condition. The current strategy is to establish a multidisciplinary team of specialists to surveil, treat, and establish continuity of care [3]. Once the diagnosis has been confirmed, referral to other specialists should be based on the previously reported complications. Nutritionist consultation is essential in cases with food-seeking behavior and rapid-onset obesity, though these patients will benefit the most from strict supervision and barriers preventing access to food. Neurology should evaluate and perform EEG, if indicated, given the increased incidence of abnormal findings in these patients [4]. Though congenital anomalies are rare, congenital heart disease is among the most common and warrants an evaluation by cardiology with an echocardiogram (ECHO). Endocrinology referral for disorders of growth and metabolism is also pertinent. Ophthalmology evaluation for detection and treatment of vision impairment should be considered. A referral to a geneticist requires consideration as patients will benefit from genetic counseling. Complete pulmonary function testing and polysomnography evaluating for respiratory and sleep disorders associated with obesity, such as obstructive sleep apnea or obesity-hypoventilation syndrome, are highly recommended. A speech-language pathologist should assess for complications related to speech and developmental delay, especially given that ASD is noted to have high penetrance in 15q13.3 microdeletion patients [5]. Recommended subspecialists and screening are further detailed in Table 2.

**Table 2:** Recommended multidisciplinary approach to screening and evaluating those with 15q13.3 microdeletion. EEG: Electroencephalogram.

Subspeciality	Screening	Evaluation
Cardiology	Evaluation for congenital heart disease such as atrial/ventricular septal defects.	Referral to a specialist for consideration of echocardiogram.
Endocrinology	Screening for metabolic disorders associated with growth impairment and obesity.	Laboratory assessment of growth hormone, thyroid-stimulating hormone, etc.
Genetics	Diagnostic testing for CHRNA7 gene mutations via whole-exome sequencing.	Referral to a genetic counselor and geneticist for diagnostic/prognostic discussion and family planning.
Neurology	Evaluation for involuntary movement and seizures.	Electroencephalogram should be considered as there is a high prevalence of abnormal EEG findings.
Nutrition	Monitoring of height/weight with the goal of maintaining 50th percentile for age.	Caloric adjustment to meet the nutritional goals. Will likely require strict supervision.
Ophthalmology	Evaluation of potential vision impairment.	Recommended yearly vision examinations.
Primary care	Early screening for the global developmental delay with an emphasis on speech and behavior.	Referral for neuropsychiatric or neurodevelopmental evaluation as indicated.
Pulmonology	Evaluation for pediatric respiratory disorders and hypoxemia.	Documentation of abnormal pulmonary sounds on examination. Record percentage of oxygen saturation and reevaluate bi-annually.
Sleep medicine	Screening of pediatric sleep disorders and nocturnal hypoxemia or obesity-hypoventilation syndrome.	Yearly diagnostic polysomnography with End-tidal CO2 (EtCO2). CPAP/BIPAP titration as needed.
Speech-Language pathology	Evaluate for speech disorders and developmental delay.	Early referral for speech and language therapy as needed.

Variations in expressivity and penetrance create challenges in establishing a prognosis even after genetic testing [6]. The lack of established treatment guidelines serves to underline the importance of continued studies and genetic testing to further establish potential mechanisms of disease development. This would serve to assist in standardizing questionnaires and genetic screening. Further studies would create opportunities to institute specific management guidelines that would focus on preventing, rather than treating, long-term complications.

## Conclusions

It is important to note that a high index of suspicion is necessary in pediatric cases of rapid-onset obesity and impulsive food-seeking behaviors to explore rare genetic disorders like 15q13.3 microdeletion of CHRNA7. A multidisciplinary care approach must be established with these patients early on to prevent future complications. The current treatment strategies aim to slow or prevent associated complications. Therapies directed at the specific genes and or receptors are in need of development. The establishment of screening questionnaires, diagnostic tools, and biomarkers are needed for early detection and intervention to avoid further comorbidities in these cases.
